# Talent Identification in Youth Soccer: Prognosis of U17 Soccer Performance on the Basis of General Athleticism and Talent Promotion Interventions in Second-Grade Children

**DOI:** 10.3389/fspor.2021.625645

**Published:** 2021-06-04

**Authors:** Andreas Hohmann, Maximilian Siener

**Affiliations:** Department of Training and Movement Science, Institute of Sport Science, University of Bayreuth, Bayreuth, Germany

**Keywords:** talent identification, talent promotion, youth soccer, child athlete, prospective study, longitudinal design, path analysis, odds ratio

## Abstract

Several talent identification programs in elementary school have implemented motor diagnostics to introduce children to groups of sports, like game sports, or even to particular sports like soccer. However, as in most other sports, in youth soccer, the predictive value of such early testing is still unclear. This prospective study evaluated the midterm prognostic validity of generic motor performance tests. The sample consisted of male second-grade children, which had received a recommendation to participate in soccer. The talent screening campaign was a basic check comprising two anthropometric parameters, five physical fitness, and three motor competence diagnostics of the German Motor Test 6–18. The test data were collected from the participating elementary school classes of the years 2010 to 2014. The soccer competition performance of those children having completed the age of at least 15 years (*n* = 502) up to the end of the season 2019/2020 (2020, September 30) was recorded. This group of U17 players was then assigned individually to five different competition levels. The prognostic validity of the physical and physiological tests was determined using ANOVAs, odds ratios, and a regression path analysis. All diagnostic methods exhibited medium-to-high prognostic validity over the 8 year time span from the talent screening to the later soccer competitions in the adolescent age groups. For later success in soccer on the province level, the 6-min run (OR = 4.28), dynamic balance (OR = 4.04), and 20-m sprint (OR = 2.46), as well as the participation in the training center of the German Soccer Federation (OR = 5.67) and the diversity of club sport activities (OR = 3.56), were of particular importance.

## Introduction

**Figure 1:**
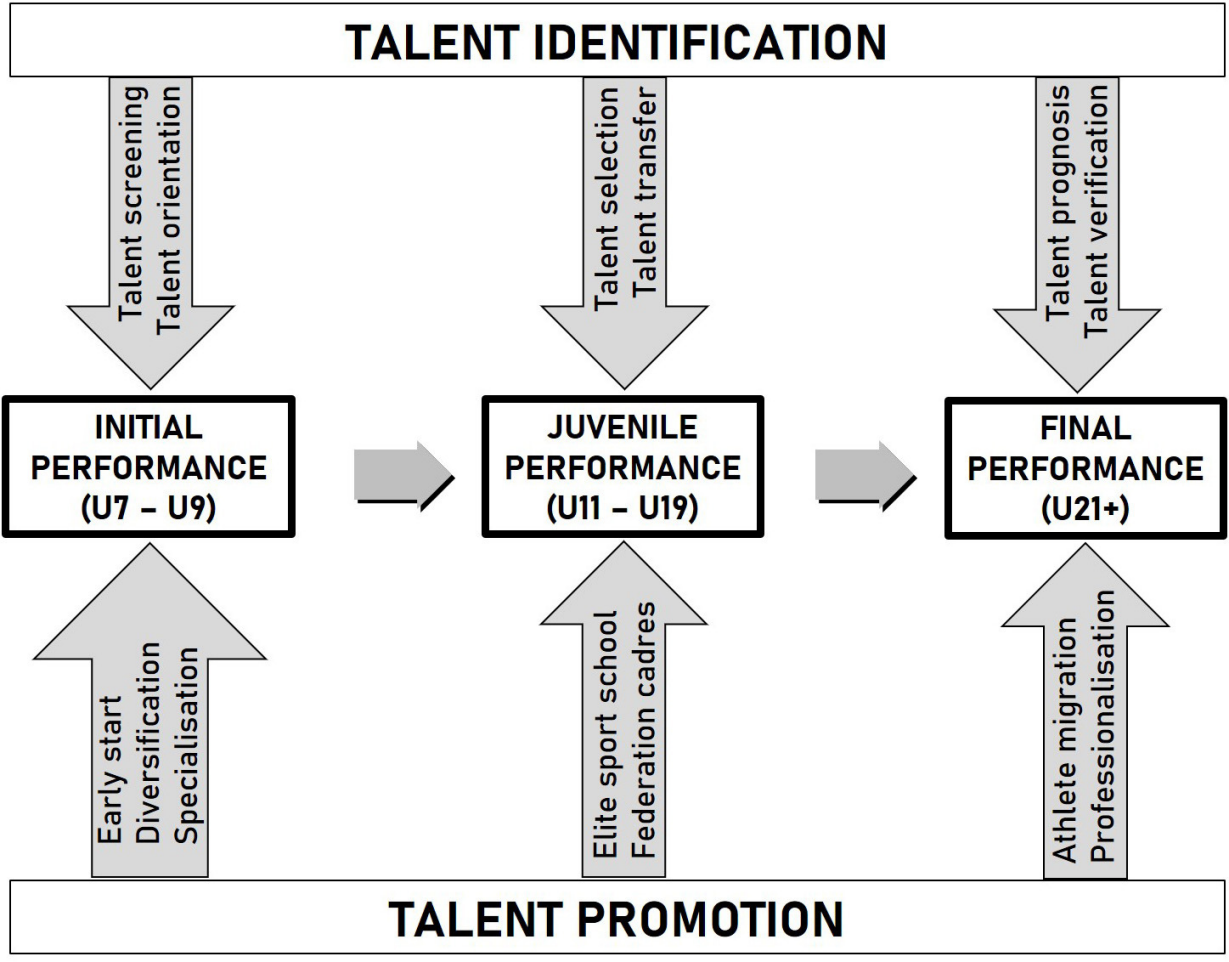
Talent identification and talent promotion as the two intertwined strategies in the long-term talent development model (mod. from Hohmann and Seidel, 2003).

Long-term talent development programs combine a diagnostic talent identification process with a sustainable talent promotion strategy (see Figure 1). On the diagnostic side of such a talent development model, early *talent orientation* includes early talent testing measures as well as recommendations for specific sports that match best with the individual ability profile. The term talent orientation is related to early talent detection in preferably untrained and thus still heterogeneous samples and “aims at motivating youngsters to choose a sport that matches the individual talent characteristics” (Pion, 2015, p. 22). Following this idea, several talent screening programs in elementary school have implemented motor diagnostics (e.g., Fuchslocher et al., 2011; Golle et al., 2015; Pion, 2015) to orientate the best movers according to the strengths of their individual profile of the general motor giftedness into specific sports where they can transform their physical, physiological, and psychological gifts through a long-term process of diligent learning, deliberate practice, and an extended amount of high-quality training into ultimate achievement (Davids and Baker, 2007; Pion, 2015). The better the individual talent characteristic profile fits to the (future) soccer demands, the higher the chances that the soccer beginners will achieve success and satisfaction in this complex team sport. This assumption is underlined by Suppiah et al. (2015), who state that a wrong choice can never be compensated by training. Engaging in an unsuitable sport might not only be detrimental to fun but also lead to dropout ahead of time. Furthermore, soccer is one of the sports that require early talent orientation (Papic et al., 2009), as on the one hand, a high amount of learning time is needed for the specific technical skills, and on the other hand, the athletes competing on the highest level got younger over the last decade. Thus, besides a systematic long-term athletic development, early talent orientation is of great relevance. In addition to that, an early talent orientation and soccer education starting at the elementary-school age gives the coaches a longer observation period and in this way reduces selection errors during early adolescence. Although most of the soccer federations administer their talent selection campaigns at a later point of time, when youth athletes have already trained for some years and developed into more homogeneous samples (Pion, 2015), early talent orientation at elementary-school age could contribute positively to the ongoing debate in talent research. In this debate, some academics warn against talent selection procedures that are conducted too early (Meylan et al., 2010), whereas others acknowledge that these selections are worthwhile to help the sport federations to focus their resources on the most talented young athletes (Unnithan et al., 2012; Zuber et al., 2015, 2016; Hoener and Votteler, 2016). The latter is especially important in soccer, as this very professional sport attracts the majority of children already at elementary-school age. Especially, if the final step of the scientific verification of the former talent prognosis was included in the talent development program of the federations, an early talent orientation could lead to a more successful talent development model in soccer.

In general, there is a lack of research investigating the prognostic value of different performance prerequisites over the full long-term period from child to adult training (Johnston et al., 2018; Sarmento et al., 2018; Williams et al., 2020). Most of the research concentrated on the middle stages of the juvenile performance development. Although previous studies questioned juvenile success even at the advanced stage of late adolescence as an appropriate indicator for soccer success in adulthood (Guellich, 2014), from recent studies on the early part of the juvenile training stage between early and late adolescence, there is compelling evidence that early talent orientation is a fruitful endeavor. So in the age group of under-15 (U15) soccer participants, various authors (Le Gall et al., 2010; Carling et al., 2012; Deprez et al., 2015; Hoener and Votteler, 2016; Hohmann et al., 2018; Sieghartsleitner et al., 2018) investigated prognostic periods of at least 3 years. Doing so, Hoener and Votteler (2016; see also Hoener et al., 2017) could show that even on the homogeneous level of the *German soccer competence centers*, a soccer-specific test battery (German Soccer Federation, 2009) provides prognostic valid and also practically worthwhile information about the 7.2 times better odds of the fastest and technically best third of the preselected players to reach the U15 junior national team. Including psychological characteristics, Zuber et al. (2016) showed that 12- to 15- year-old individuals having started early with soccer training had 2.5 times better chances to reach the Swiss U17 national team than players starting later in time with soccer-specific deliberate training.

Although reliable and valid information about the future potential of talented players on the basis of motor abilities and technical skills diagnostics is a valuable tool in talent development programs for sport clubs and federations, several studies question the long-term predictability of future success (Cote et al., 2009; Pankhurst and Collins, 2013; Carling and Collins, 2014). The main reasons for these scientific concerns arise from the often undifferentiated mixture of generic as well as sport-specific tests in talent identification campaigns, poor operationalization of the criterion variables, and the unsystematic timing of cross-sectional diagnostics at single points in time during the long-term athletic development process (Bergkamp et al., 2019). It thus comes as no surprise that the great variety of study design parameters have led to inconsistent research results, providing an ambiguous picture with regard to the prognostic validity of physical and physiological tests addressing generic motor abilities and sport-specific technical skills. Especially in the adolescent U15 age group, some studies verified a medium stability (Deprez et al., 2015) and prognostic validity of physical and physiological tests (Figueiredo et al., 2009; Hoener and Votteler, 2016; Sarmento et al., 2018), whereas others did not find significant associations between test results and later success in youth, junior, or adult soccer (Le Gall et al., 2010; Carling et al., 2012). Recent talent research in soccer by Hoener and Votteler (2016; see also Hoener et al., 2017) and Hohmann et al. (2018) has made clear that a longitudinal investigation of motor predictors' prognostic relevance for long-term success is not only a key topic in talent research (see also Gonaus and Mueller, 2012; Sarmento et al., 2018; Bergkamp et al., 2019) but also an indispensable prerequisite for a sophisticated understanding of the changing prognostic validity of the various diagnostics at the different stages of the non-linear and multifactorial long-term development of elite soccer players (Skorski et al., 2016).

As soccer is based on a complex, multidimensional performance profile (Williams and Franks, 1998; Reilly et al., 2000; Williams and Reilly, 2000; Huijgen et al., 2014; Buekers et al., 2015; Forsman et al., 2016b; Johnston et al., 2018, the early talent screening is by definition focused on a multifaceted variety of general physical, physiological, and psychological performance diagnostics in heterogeneous populations. With this in mind, our study focused on general anthropometric, physical fitness (PF), and motor competence (MC) characteristics. PF can be defined as the overall performance in various strength, speed, and endurance tasks in a specified physical, social, and psychological environment (Demetriou et al., 2019). MC summarizes the degree of proficiency in a wide range of motor tasks as well as the movement quality, coordination, and control leading to a particular motor performance (Bardid et al., 2019). The generality–specificity dilemma says that the testing of soccer-specific skills is not yet suitable for beginners like the second-grade elementary-school children at hand. The reason is that even the best movers do not yet possess the high-fidelity skills of dribbling, passing, or shooting (Hohmann et al., 2018). Notwithstanding this, Pion et al. (2020) assume that even on the basis of a generic test battery, it is possible not only to identify sporting potential in primary school but also, on the one hand, to orient children toward a sport that best suits their personal strengths or, on the other hand, to facilitate the transfer between similar sports. However, the authors concluded that more research is needed on how to assist children in choosing sports that match their own traits and preferences. Thus, the aim of this long-term study is to examine the prognostic validity of a more fundamental and general testing of early athleticism on the second-class age level, although this cohort included some older scholars who had to repeat the second class in the year 2010, with 93.1% of the vast majority of the test participants representing the U9 age group. So for the sake of easier communication in the following, we adhere to the age category U9 to refer to the soccer-specific age group framework.

The present study is a follow-up version of a previous study of Hohmann et al. (2018) to examine the prognostic validity of a broader performance testing at the same age of second grade of elementary school. In contrast to the former study, which investigated prognostic validity of 11 general performance characteristics and two soccer-specific skill tests (agility run and soccer dribbling) over a midterm follow-up period in regard to the U15 age group, in the current study, the follow-up period was extended to a long-term prognosis until the U17 age group. In this study, the test results of the investigated second-class children in two anthropometric, five PF tests, and three MC tests are compared with their successes as juvenile soccer athletes at age U13 and U17. By including the intermediate U13 stage of the participants' soccer performance, this study allows for a more complex picture of the soccer performance development. Athletes who later show better performances should also be able to be distinguished significantly from later weaker athletes already in the U9 age group. By means of this prospective cohort design, this study is aimed on the extension of the current knowledge on the prognostic validity of very early talent characteristics, as the majority of previous studies focused on the later stages of the U12 and U15 age groups.

## Materials and Methods

### Study Design

**Figure 2:**
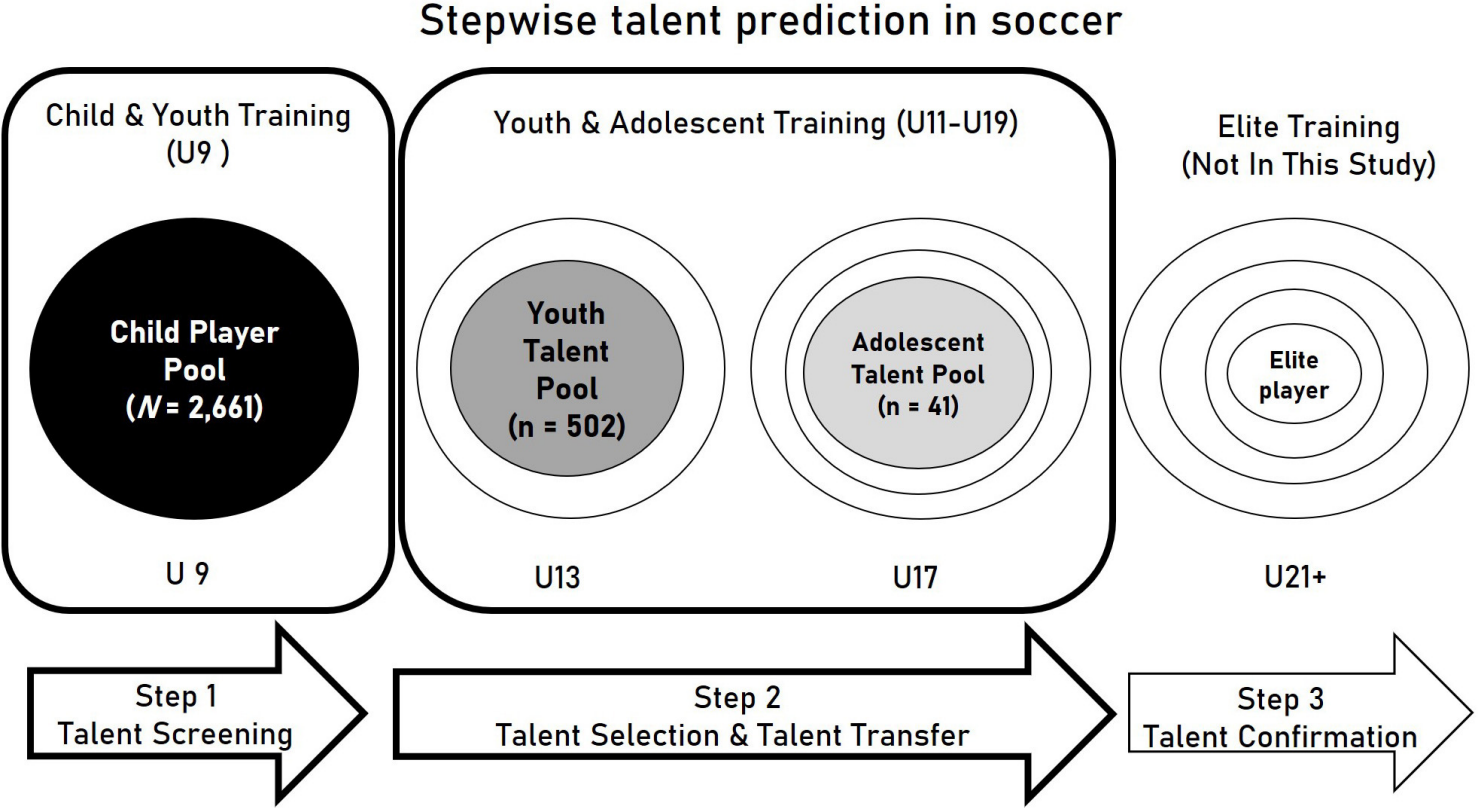
Stepwise talent prediction during the three stages of the talent development process.

According to our model of long-term talent development (see Figure 1), the relevance of the performance characteristics profile has to be investigated in a stepwise procedure for at least three prognostic periods from the beginning of a talent development program until the full reach of the professional level as its final destination (Figure 2). Each prognosis should allow for midterm soccer performance predictions over a time span of about 5 years (Hoener et al., 2017). As talent development in soccer mostly starts in the U9 age group during elementary-school attendance, the youth training center of the federation starts at age 12 and promotes the talents until age 15. Besides the youth training centers of the soccer federation, the professional soccer clubs run certified soccer academies including players of the same age and also older ones of the age group U17. The final stage of elite adult expertise is reached earliest at about age 21; and in most cases around the age of 23 years, the long-term perspective should cover a development period of about 15 years. This also corresponds to the average time span of 15 years that is needed to reach international soccer excellence (Leite et al., 2009).

The second-grade scholars' physical and physiological test results served as predictors for the participants' later success in soccer at the intermediate point in time of age U13 and finally in late adolescence, which is the U17 age group. The total prognostic time span between the testing and the adolescent competition U17 success was *M* = 7.94 years (*SD* = 0.87).

University staff members and students conducted the testing in all five test campaigns (2010–2014). After the testing, the participation of the children in soccer competitions on any youth soccer division level was recorded from the beginning of the season 2010/2011 (2010, September 30) until the end of the season 2019/2020 (2020, September 30). The data were extracted from the regional print media (tournament and soccer match lists), the official DFB website (www.fussball.de; www.torgranate.de), and the official team lists provided by the soccer clubs.

Before entering the test campaign, all children's parents provided written informed consent for the recording and scientific use of the data collected in the anthropometric and motor tests. The university's ethics department approved the implementation of this study.

### Participants

**Figure 3:**
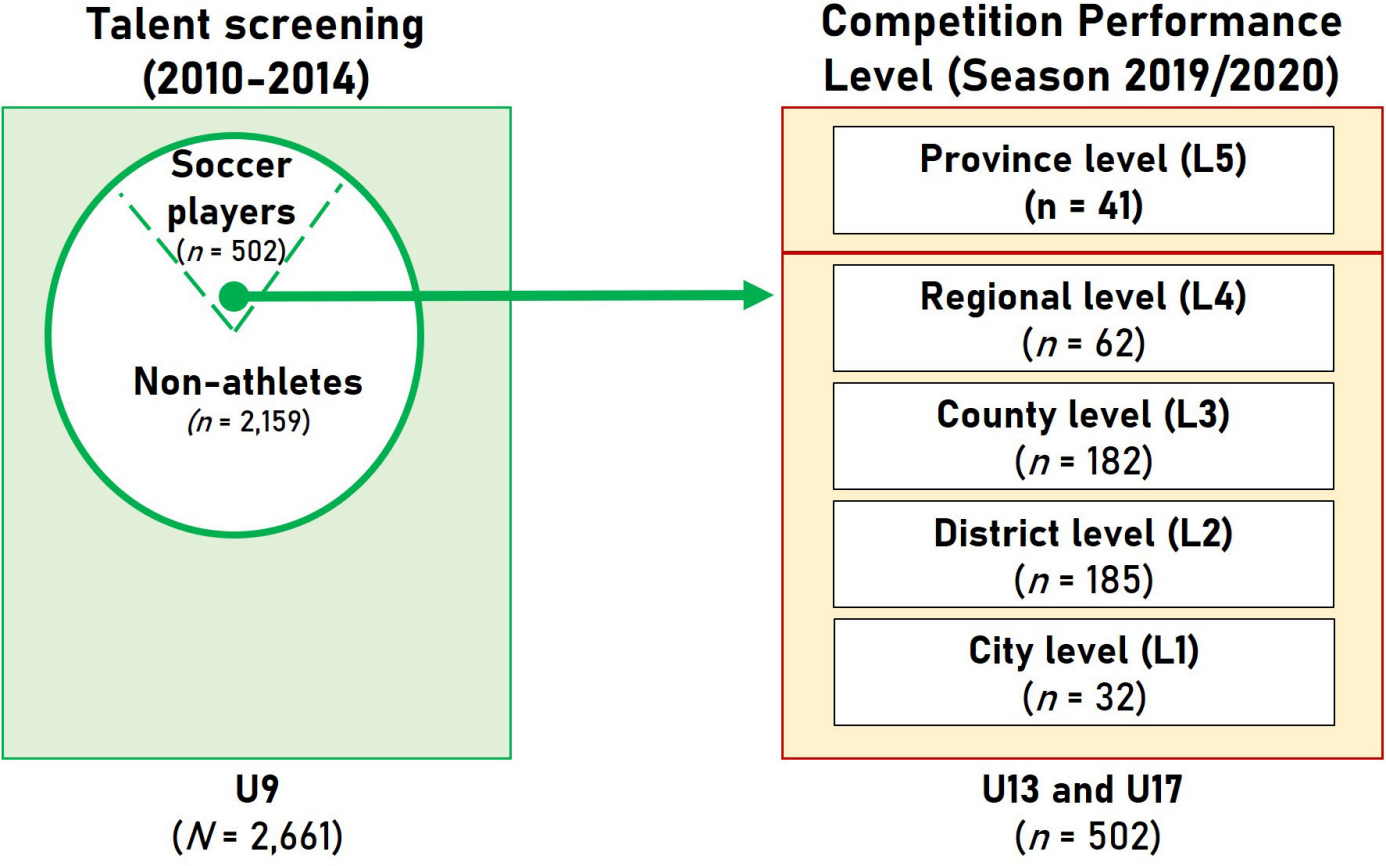
Scheme of the participants' groups of the talent screening campaign (2010–2014) included in this study.

All soccer participants underwent the motor diagnostics at the average age of *M* = 8.10 years (*SD* = 0.73) together with their classmates who did not engage in youth soccer. For the prospective cohort study at hand, we investigated the data from *N* = 2,661 male second-grade children with no more than one test data missing. All 2,661 study members took part in the talent screening campaign in the years 2010–2014. Out of this total population, *n* = 502 were found to compete later on in official soccer games independent from the playing division and also reached the age of 15 years (≥180 months) until the deadline of the sport season 2019/2020 (2020, September 30; Figure 3). Children who had chosen other competition sports other than soccer were not included in this study to allow for the comparison of the soccer participants with actually untrained children.

### Measures

#### Performance Characteristics

Besides the two physical characteristics body height and body weight, the testing included five physiological fitness, and three MC tests, which were used as performance predictors. The test tasks aimed at the diagnosis of sprint, coordination, balance, flexibility, arm and upper body strength, leg power, ball throw, and endurance performance. The standardization of the test items was secured by protocols, which included a detailed description of test materials and setup, test demonstration, warm-up and training trials, assessment, and registering of the test scores (Boes and Schlenker, 2016). Besides the generic motor tests, each player's body height, body weight, and calendar age (measured by the month of birth within a calendar year) were registered.

##### 20-m Sprint

Time for a 20-m linear running sprint (Boes et al., 2001). The run time was measured by means of light gates (Brower Timing Systems; Draper, USA). The starting position was 0.3 m behind the start line. Between the two possible attempts, a break of at least 2-min was kept. The reliability of this test is *r*_tt_ = 0.90 (Boes and Schlenker, 2016). For easier understanding, in the following, we changed the scale into positive direction for faster run times.

##### Sideward Jumping

The test contains of 15 s of sideward jumping within two adjacent 50 × 50 cm squares (Kiphardt and Schilling, 1970). The number of two-legged jumps from one square to the other without touching a boundary line was measured. Five trial jumps were allowed before the testing began. Between the two possible attempts, a break of at least 2-min was kept. The objectivity of this test is *r*_obj_ = 0.99, and the reliability is *r*_tt_ = 0.89 (Boes and Schlenker, 2016).

##### Balancing Backwards

Balance backwards on 6-, 4.5-, and 3-cm wide beams (Kiphardt and Schilling, 1970). For each bar, the number of steps backwards balanced (feet fully raised) until leaving the bar was counted. The maximum number of steps per attempt was limited to eight. For each of the three beams, two possible attempts were made. Thus, the result was the sum of all steps taken (maximum: 48 steps). There was a short practice period before the test was carried out. The objectivity of this test is *r*_obj_ = 0.99, and the reliability *r*_tt_ = 0.73 (Utesch et al., 2015).

##### Standing Torso Bend Forward

A standing torso bend forward test was performed as a flexibility test (Fetz and Kornexl, 1978). Here, the participants tried to reach as far as possible with their fingertips beyond their feet and to hold this position for at least 3 s. The distance of the fingers in cm to ground level was recorded, whereby a low range aboveground level was recorded as a negative distance. There were two attempts allowed. The objectivity of this test is *r*_obj_ = 0.99, and the reliability *r*_tt_ = 0.94 (Boes and Schlenker, 2016).

##### Push-Ups

The push-up test was carried out after a short trial period. Within 40 s, the number of fully completed repetitions is counted. Hands have to touch each other when the body is lying down on the floor and also when the arms are extended after the push-up. A complete repetition was only evaluated when the upper body was laid down on the mat and hands touched each other (Haag and Singer, 1981). Only one attempt was carried out here. The objectivity of this test is *r*_obj_ = 0.98, and the reliability *r*_tt_ = 0.69 (Boes and Schlenker, 2016).

##### Sit-Ups

Similar to the push-up test, the time available for the push-up test was limited to 40 s (Boes et al., 2001). After a short practice phase, only one test was granted, and the number of correctly executed sit-ups was counted. The objectivity of this test is *r*_obj_ = 0.92, and the reliability *r*_tt_ = 0.74 (Klein et al., 2012).

##### Standing Long Jump

The standing long jump was carried out without a previous tryout. The distance of the two-leg standing jump was measured in cm (measured from the heel; Fetz and Kornexl, 1978). A break of at least 2-min was observed between the two possible attempts. The objectivity of this test is *r*_obj_ = 0.99, and the reliability *r*_tt_ = 0.89 (Boes and Schlenker, 2016).

##### 6-min Endurance Run

A 6-min endurance run around a volleyball pitch (9 × 18 m) was carried out (Fetz and Kornexl, 1978). There, the number of meters covered was measured. The test was conducted in groups of 15 persons at the same time. The reliability of this test is *r*_tt_ = 0.92 (Boes and Schlenker, 2016).

All players were assessed under similar conditions. The tests were carried out during regular school hours (8–12 pm) by qualified test personnel. The testing always began after a uniform warm-up phase with the 20-m sprint and ended with the 6-min endurance run. In all tests, except for sideward jumping (where the average of the two attempts was taken as test result), the better one of the attempts counted.

All eight tests were examined in a whole series of studies by various authors (Klein et al., 2012; Utesch et al., 2015; Boes and Schlenker, 2016) with regard to the test standards. Boes and Schlenker (2016) analyzed the test battery's psychometric properties for a sample consisting of nearly 50,000 school children and adolescents. The authors found an average test–retest correlation coefficient of *r*_tt_ = 0.82 at elementary-school age (7–11 years), even if there is considerable variation between the tests ranging from *r*_tt_ = 0.52 for balancing backward to *r*_tt_ = 0.94 for the bend forward.

#### Talent Promotion Interventions

On the side of the talent promotion variables, the club sport diversity of the 502 soccer participants was quantified by the number of different club sport activities with at least one official competition participation attended voluntarily before or in parallel to the talent screening, as well as during the following period of soccer practice during youth age.

Also very early, the sport administration of the government of the Hessen province offers a local talent promotion course, which is conducted by an expert sport teacher. Out of the 502 soccer participants, about 5% (*n* = 26) of the players took part in this talent promotion program. Such an additional 90-min school sport session takes place once a week and is open to about 10–15% of selected pupils with the aim to enhance the basic components of PF and MC. Thus, the generic training program aims primarily at the development of fundamental movement skills (Burrows et al., 2014; Barnett et al., 2016). The talent promotion courses start during the first grade of elementary school (normally at the age of 6–7 years) and end with finishing elementary school after the fourth grade.

As a third talent promotion strategy, two certified schools from the tested region offer sport-specific talent promotion classes, which were attended by about 10% (*n* = 51) of the soccer players. The sport classes consist of about 25 children who are selected and intensively guided by expert school sport officials. The membership in a sport class of one of the two local youth sport schools could comprise a maximum duration 6 years starting at the fifth grade (normally at the age of 11 years) and ending after the 10th grade (at the age of 16 years), which is 2–3 years before finishing high school education. The sport class members take part in two additional training sessions embedded in the official school timetable in the morning. Both training, sessions take place in sport-specific training centers and are guided by qualified expert coaches of the particular sport. The training focuses on general as well as sport-specific performance prerequisites.

As a fourth talent promotion campaign, the German Soccer Federation (DFB) offers between the age of 12 and 15 years 1 weekly soccer training session conducted by expert soccer coaches in one of the 366 soccer training centers distributed all over Germany. Building up on a basic soccer promotion stage (U11 groups and younger), the soccer training (or competence) centers form the second level of the pyramid of the successful talent promotion system of the German soccer system. The soccer training centers prepare about 14,000 promising youth athletes per year over 3 years in the age groups U12, U13, U14, and U15 (in some regions also U11), so the players can participate for 4 years in the program (Schott, 2010). In parallel, more than 50 professional soccer clubs run youth soccer academies, which include players from the same and also older age groups. The youth soccer academies are attached to the professional soccer clubs of the first, second, and third professional soccer divisions (Bundesliga). The youth soccer academies represent the second level of the talent promotion of the DFB and open the door to the third and final step to participate in the different DFB youth national teams. About 8% (*n* = 40) of the investigated soccer players were selected for a membership in one of the two regional talent promotion centers, with four players transferring to different youth soccer academies at age 15 after finishing with the training center.

#### Competition Performance

The soccer performance level (Table 1) reached by the soccer players until the end of the season 2019/2020 was utilized to quantify all athletes' success in early adolescence as criterion variable. All second-grade athletes who participated in the motor diagnostics and participated a minimum of one official soccer competition in a division or city level until the end of the season 2019/2020 or at least held an official club license without taking part in competitions (level 1) were recorded. Based on their success in the youth soccer competitions, the individual soccer performance was ranked from level 1 up to level 5, if the athlete was playing on the province “*Hessen level*.” Although the participation in the Hessen soccer division (level 5) was the highest level registered in this study, from a nationwide perspective, such a soccer competition participation represents only a sub-elite soccer performance level. So it has to be stressed that there is still a gap between the soccer performance investigated in this study and the nation's best soccer performances within the U17 age group. To promote especially the elite youth soccer level, the German Soccer Federation not only organizes an U17 national championship by means of a first division competition (Bundesliga) but also promotes an U17 national team for Germany's best players.

**Table 1:** Five-level scale for the recording of the soccer-specific competition results of the study participants taking part in official soccer competitions of the age groups U13 and U17 (*N* = 502).

**5-level scale of U13 and U17 age group soccer performance**		
Level 1	*N* = 32	Holder of an official soccer club license or competition participation on the city level (Kreisklasse) at the age of U13 and U17
Level 2	*N* = 185	Competition participation on the district level (Kreisliga) at the age of U13 and U17
Level 3	*N* = 182	Competition participation on the county level (Bezirksliga, Gruppenliga) at the age of U13 and U17
Level 4	*N* = 62	Competition participation on the regional level (Verbandsliga) at the age of U13 and U17
Level 5	*N* = 41	Competition participation on the province level (Hessenliga) at the age of U13 and U17

In contrast to a former study of Hohmann et al. (2018) on younger soccer players, in the U17 age group, the names of almost all study participants were individually reported in the team rosters published by the local media and the website of the Hessen Soccer Federation, thus substantially increasing the study sample of this study. However, the performance levels of game sport athletes are difficult to judge (Roescher et al., 2010; Gonaus and Mueller, 2012; Hoener and Votteler, 2016; Leyhr et al., 2020). To enhance the reliability of the assignments of the soccer players to the different competition performance levels, the records of the soccer performance of the U13 as well as the U17 age group were checked fine-grained, that is, individually player by player, by the head coach of the local competence center of the German Soccer Federation (DFB) who was in charge of the nomination, selection, and education of the youth soccer players between the age period of 12 and 15 years promoted by the DFB during the investigation period. As the scale of the five performance levels of the city, district, county, regional, and Hessen province leagues is not standardized for the different age groups during childhood and adolescence, and thus the age-specific performance levels do not accord perfectly with each other, this might impair the comparability of the individual soccer performances at the U13 and U17 age stages. Thus, in addition to the coach's individual rating, we checked the predictive validity of different age-specific soccer performances in our study sample. The high intercorrelations of the age-specific soccer performance variables (U13 ^*^ U15: *r*_tt_ = 0.79, *n* = 502, *p* < 0.001; U13 ^*^ U17: *r*_tt_ = 0.79, *n* = 502, *p* < 0.001; U15 ^*^ U17: *r*_tt_ = 0.89, *n* = 502, *p* < 0.001) underpinned not only the usability of the criterion variables but also the developmental stability of the individual pathways of the soccer performance development^1^.

### Statistical Analysis

In order to obtain solid results in regard to the prognostic relevance of the predictors, the data sets of four successive second grade cohorts (2010–2014) were collected, so the samples of the soccer players achieved a sufficient number on the higher competition levels. To gain insight into the prognostic relevance of the 10 predictors (two anthropometric variables and eight motor tests) in the soccer group and to clarify their predictive validity for the single dependent (criterion) variable of the adolescent soccer performance at age U17, univariate ANOVAs were conducted, analyzing mean differences between the five different soccer performance levels. According to Cohen (1988), the magnitude of effects was as classified small (η^2^ = 0.01), medium (η^2^ = 0.06), and large (η^2^ = 0.14).

For the evaluation of motor predictors' prognostic validity, the age influence on the test performances should be considered (Meylan et al., 2010; Carling and Collins, 2014; Hoener and Votteler, 2016; Hoener et al., 2017). So ANOVAs were conducted to check the data set for significant differences of the youth athletes in regard to the yearly birth quarter. To control for confounding effects of the systematic influence of age found in the tests sideward jumping, 20-m sprint, push-ups, sit-ups, and 6-min run, the calendar age (in months) was partitioned out of the results in all 10 predictors by bivariate regression analysis. In the bivariate regression analyses, the test results served as the dependent variable, and the age (in months) as the independent variable (Siener and Hohmann, 2019). Furthermore, to allow for comparisons of the soccer-specific relevance of the different predictors and to estimate the lead of the soccer athletes against the general age group population, all test data were standardized by *z*-values based on the mean value and standard deviation data of the untrained athletes.

On the side of the talent promotion variables, the (i) club sport diversity was quantified by the number of club sport activities with an official competition participation before or in parallel to the testing and practicing soccer afterwards and thus represented a discrete variable. The participation in a (ii) local talent promotion course offered by the Hessen government sport administration, the attendance of a (iii) sport class of one or both local youth sport schools, and the membership in the (iv) local soccer training center of the German Soccer Federation (DFB) represented dichotomous dummy variables. Whereas, 411, that is, 81.9% of the *N* = 502 study participants, did not take part in any of the three talent promotion measures (ii–iv), 70 children (13.9%) participated in one of the interventions. From the remaining 21 (4.2%) of the investigated youth athletes, 12 of the 51 sport school members exercised also in the local soccer training center, but only seven of the sport school members took part in the governmental talent promotion course. Furthermore, four members of the local soccer training centers visited the governmental talent promotion courses in parallel. None of the scholars participated in all three talent promotion programs.

According to former test validation results in youth soccer athletes (Hohmann et al., 2018) and a coach survey including 32 highly qualified soccer coaches ranking the eight test according to their relevance for youth soccer performance, a soccer recommendation score (SRS) was calculated for each participant. The SRS was formed by the mean value of a selection of the five most influential and, respectively, highest ranked general performance prerequisites, thus being a more complex soccer performance predictor than the single tests *per se*. On the basis of the ranking results of the previous studies, each of the five selected tests was hierarchically weighted by a stepwise weight factor (WF) between 1.2 and 2.0 according to their estimated validity for soccer performance. Thus, the 20-m sprint (WF 2.0), sideward jumping (WF 1.8), 6-min run (WF 1.6), standing long jump (WF 1.4), and sit-ups (WF 1.2) were used, because they had turned out to be more soccer-related than the other three tests and the two anthropometric measures. Based on this weighting, the SRS was calculated according to Formula (1).

(1) $$\begin{array}{r} SRS=2^{*}z_{20m\;sprint}+1.8^{*}z_{sideward\;jumping}+1.6^{*}z_{6min\;endurance\;run}\\ \;+1.4{\;}^{*}z_{standing\;long\;jump}+1.2^{*}z_{situps} \end{array}$$

For a clearer view of a player's relative chances depending on several predictors, odds ratios (ORs) for the *z*-standardized performances were computed. On the basis of the OR, the prognostic validity of the test results was obtained. Furthermore, the questions, how many non-talented athletes would be disqualified (specificity in regard to true negatives), and how many talented youngsters would be successfully identified (sensitivity in regard to true positives) by the test battery were analyzed by calculating the odds stepwise between the 0.5% and up to 99.5% rank thresholds in the *z*-standardized test performances (Hoener and Votteler, 2016). To determine the optimal relation between drafted talents and excluded non-talents, the Youden index (also *Youden's J*) was calculated. This figure ranges between 0 (random result) and 1 (optimum result) and is calculated by summing up the two parameters sensitivity and specificity. Youden's *J* allows to quantify the prognostic validity independent of the sample size of the two performance groups (Youden, 1950). In this study, the basic assumption of Youden's *J* that specificity and sensitivity are of equal importance is fulfilled. Unlike in the soccer federations training centers or in sport school classes, at elementary-school age, there is still sufficient capacity in the soccer clubs to promote all promising youngsters. Thus, there is no need to reduce the number of false-positive soccer recommendation. Also, an increase of the number of true positives has not to be preferred one-sided, as almost all male second-class participants in school get in touch with the sport of soccer anyway and thus could be still detected as soccer talents by teachers and the soccer coaches later on.

A path-analytical model based on stepwise multivariate regression analyses examines the causal pathways of influence of independent variables on a dependent criterion variable measured later in time (Bryman and Carter, 1994). Thus, the influence of the 10 performance characteristics assessed at second grade, as well as the influence of the four different talent promotion interventions (diversity of club sport activities besides soccer, participation in the governmental talent promotion program, attendance of a sport school class, and membership of the German Soccer Federation training center) on the later soccer performance, was determined here. In a first step of the regression path analysis, all 10 performance characteristics (eight test performances and two anthropometric measures) and the four talent promotion interventions, plus the soccer performance level at age U13, were included as predictors, and their influence on the criterion variable performance level at age U17 was evaluated. In a second step, the soccer competition performance at age U17 was eliminated from the next regression analysis, and now the formerly independent early soccer competition performance at age U13 served as the dependent criterion variable.

In all procedures, data were analyzed using SPSS version 25.0 (IBM Corp.), and minimum level of significance was set at *p* < 0.05.

## Results

### Test Performances of the Soccer Players and the Non-athletes

The descriptive performance characteristics of the test participants are presented in Table 2.

**Table 2:** Descriptive statistics of age, soccer competition performances, two anthropometric, and eight motor diagnostics, soccer recommendation score, and four different talent promotion interventions in second-grade children taking part in the test campaigns 2010–2014 and competing in youth soccer competitions later on in the age groups U13 and U17 until the end of the season 2019/2020.

**Variables**	**Groups**	***N***	**M**	***SD***	**SE**	**Min**	**Max**
**Calendar age**							
U9: Calendar age (months)	Soccer players	499	95.23	7.24	0.21	82.00	125.00
	Non-athletes	2,159	95.53	6.48	0.14	75.00	127.00
U13: Calendar age (months)	Soccer players	501	148.74	7.70	0.24	156.00	179.00
U17: Calendar age (months)	Soccer players	502	192.47	7.94	0.36	180.00	203.00
**Soccer performance**							
U13: Performance level (pts)	Soccer players	501	1.89	0.78	0.03	1.00	5.00
U17: Performance level (pts)	Soccer players	502	2.80	1.02	0.05	1.00	5.00
**Performance characteristics**							
Body height (cm)	Soccer players	490	129.74	5.63	0.25	110.00	147.00
	Non-athletes	1,997	129.45	6.27	0.14	107.00	154.00
Body weight (kg)^†^	Soccer players	490	28.08	4.89	0.22	18.30	54.80
	Non-athletes	1,997	28.69	6.11	0.14	16.40	70.004.70
Sideward jumping (reps)^†^	Soccer players	493	25.55	6.14	0.28	11.00	44.00
	Non-athletes	1,993	23.07	6.40	0.14	0.50	44.50
Balancing backwards (steps)^†^	Soccer players	493	29.76	8.69	0.39	7.00	48.00
	Non-athletes	1,994	26.42	8.98	0.20	3.00	48.00
Standing long jump (cm)^†^	Soccer players	491	135.78	17.05	0.77	70.00	181.00
	Non-athletes	1,983	125.36	18.86	0.42	57.00	193.00
20-m sprint (s)^†^	Soccer players	493	4.50	0.33	0.01	3.54	5.58
	Non-athletes	1,992	4.60	0.39	0.01	3.60	7.15
Push-ups (reps)^†^	Soccer players	493	14.09	4.09	0.18	1.00	26.00
	Non-athletes	1,992	13.24	3.84	0.09	0.00	26.00
Sit-ups (reps)^†^	Soccer players	493	20.06	5.22	0.23	0.00	35.00
	Non-athletes	1,992	17.51	5.69	0.13	0.00	35.00
Bend forward (cm)^†^	Soccer players	488	0.87	5.25	0.24	−19.50	15.00
	Non-athletes	1,981	−0.54	6.06	0.14	−23.00	20.00
6-min run (m)^†^	Soccer players	481	1,019.68	120.40	5.49	604.00	1,359.00
	Non-athletes	1,953	907.31	133.51	3.02	108.00	1,306.00
**Soccer recommendation score**							
Soccer recommendation score (*z*-value)^†^	Soccer players	481	−0.29	1.14	0.05	−4.62	3.00
**Talent promotion interventions (U17 soccer performance)**							
German Soccer Federation training center (pts)^*^	Members	40	3.98	0.80	0.13	3.00	5.00
	Non-members	462	2.70	0.98	0.05	1.00	5.00
Sport school class (pts)^*^	Members	51	3.40	1.05	0.15	1.00	5.00
	Non-members	451	2.73	1.00	0.05	1.00	5.00
Governmental talent promotion program (pts)^*^	Members	27	3.27	1.04	0.20	1.00	5.00
	Non-members	475	2.77	1.02	0.05	1.00	5.00
Club sports diversity (pts)^ns^	High diversity (≥3)	14	3.21	1.05	0.28	2.00	5.00
	Low diversity ( ≤ 2)	488	2.79	1.02	0.05	1.00	5.00

† *Significant between soccer players and non-athletes*;

* *significant between members and non-members*;

ns *not significant between high and low club sports diversity*.

**Figure 4:**
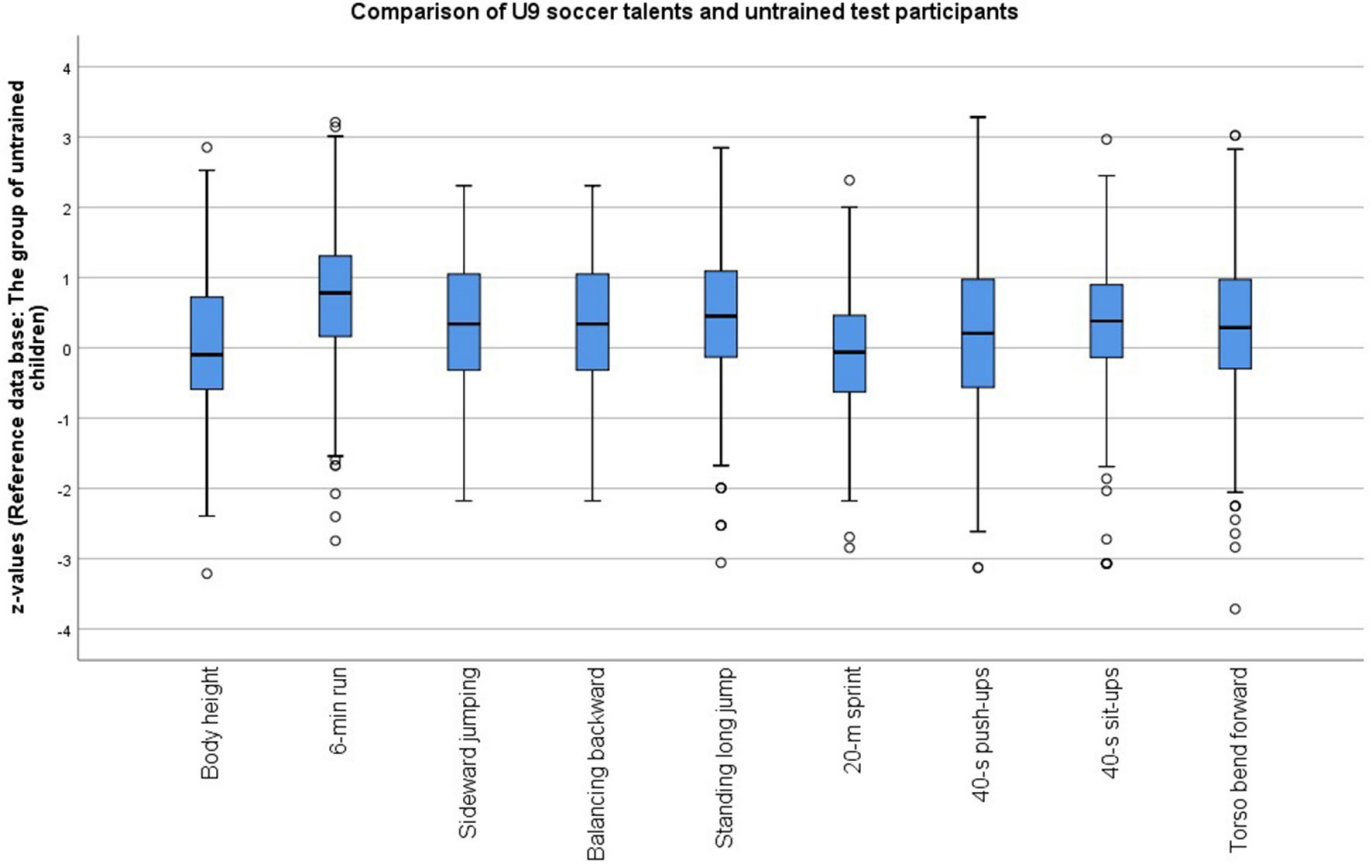
Age-adjusted and *z*-standardized test performances of the soccer players and non-athletes of the second grade of elementary school in the test campaigns 2010–2014.

The early talent screening results for the age-adjusted and *z*-standardized performance characteristics in Figure 4 demonstrate in nine of the 10 diagnostics better performances in the soccer players compared with the non-athletes who served as the reference group (*M*_z_ = 0). As described above, the five test results of the 20-m sprint, sideward jumping, 6-min run, standing long jump, and sit-ups formed the basis of the SRS. The adequacy of this selection is corroborated by the result that the soccer group exhibited already in the talent screening campaign in the second grade especially in these tests much better soccer-oriented performance prerequisites than did the non-athletes.

### Predictive Validity of the General Performance Characteristics in Soccer

#### ANOVAs and Odds Ratios

**Figure 5:**
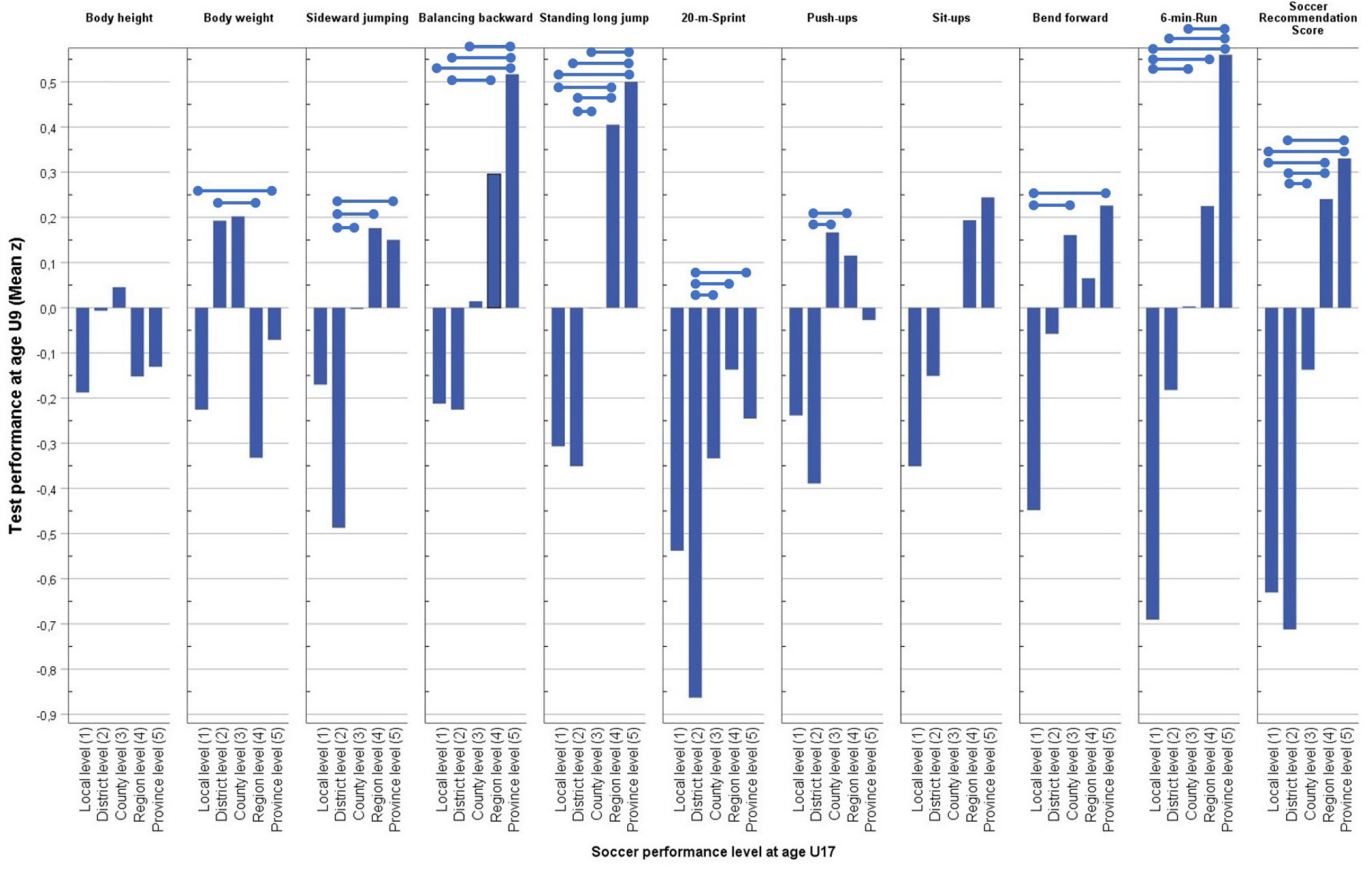
Age-adjusted and *z*-standardized test performances of the U9 participants in regard to the later competition performance levels at adolescence (U17). Data of the 20-m sprint were transformed into positive direction. Variances of subgroups were homogeneous, but not in body weight [Levene (4;488) = 5.39; *p* < 0.001]; significant differences (Bonferroni *post-hoc* test; *p* < 0.05) between subgroups are marked by connection lines).

Within the group of the U9 soccer players (*n* = 502), the ANOVAs demonstrated significant differences in the performance characteristics, which were in line with the differences in the later performance levels reached by the children as adolescents in the U17 age group. Soccer players who reached higher juvenile performance levels (see Figure 5) performed better not only in the SRS [*F*_(4;476)_ = 16.36; *p* < 0.001; η^2^ = 0.11] but also in all eight single motor tests: 20-m sprint [*F*_(4;491)_ = 12.06; *p* < 0.001; η^2^ = 0.09], standing long jump [*F*_(4;489)_ = 10.95; *p* < 0.001; η^2^ = 0.08], sideward jumping [*F*_(4;491)_ = 9.15; *p* < 0.001; η^2^ = 0.07], 6-min run [*F*_(4;479)_ = 8.77; *p* < 0.001; η^2^ = 0.07], push-ups [*F*_(4;491)_ = 7.79; *p* < 0.001; η^2^ = 0.06], balancing backward [*F*_(4;491)_ = 6.96; *p* < 0.001; η^2^ = 0.05], bend forward [*F*_(4;486)_ = 3.90; *p* < 0.01; η^2^ = 0.03], and sit-ups [*F*_(4;491)_ = 3.27; *p* < 0.05; η^2^ = 0.02]. In regard to the two anthropometric measures, the soccer players' body weight corresponded negatively to the individual performance level [*F*_(4;488)_ = 4.43; *p* < 0.01; η^2^ = 0.03], whereas the body height [*F*_(4;488)_ = 1.08; *p* = 0.366; η^2^ = 0.006] did not go systematically hand in hand with the later-on soccer performance level in the U17 age categories.

**Figure 6:**
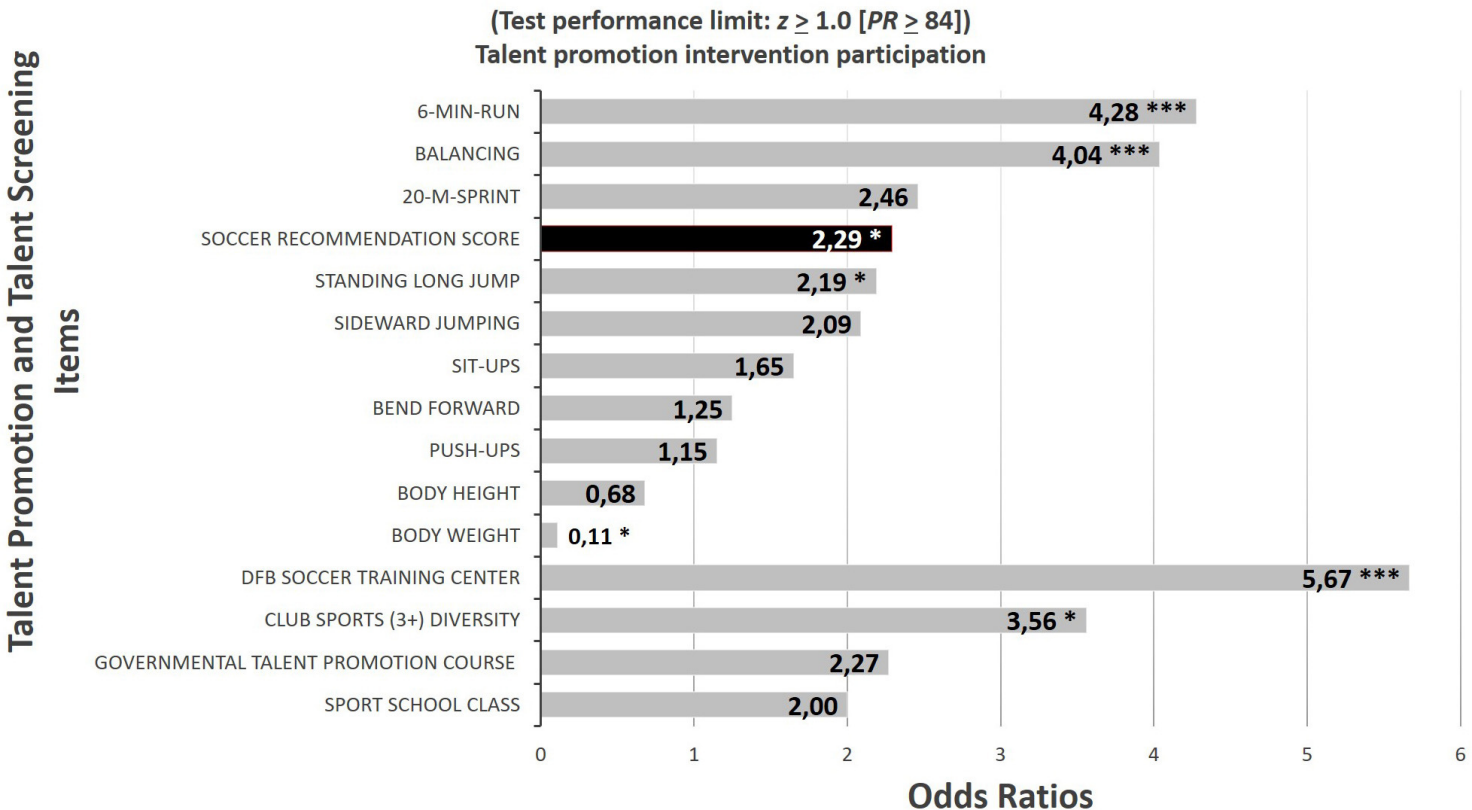
Odds ratios (at the *z* = 1.0 and *PR* = 84 threshold) of the two body dimensions and eight generic motor tests and the soccer recommendation score, as well as of four talent promotion interventions to predict the relative chances of the U9 participants to reach the highest (province) level of soccer competition performance at adolescent age (U17). Significant results are marked by asterisks: *p < 0.05; ***p < 0.001.

ORs for each single test as well as for the SRS represent the prognostic validity of the investigated predictors and make the sport-specific relevance of the different tests comparable. In the context of this study, the ORs quantify the relative chances of an U9 participant to reach the performance level 5 until the adolescent age group U17. In Figure 6, the ORs were calculated for participants who had achieved an individual test score among the best 16% of the total group (*z* ≥ 1.0, corresponding to *PR* ≥ 84) in the SRS, and in any of the eight single tests and the two body dimensions. With the use of this cutoff limit, the ORs of the performance characteristics 6-min run (*OR* = 4.27; *X*^2^ = 19.74; *p* < 0.001), balancing (*OR* = 4.03; *X*^2^ = 18.08; *p* < 0.001), and standing long jump (*OR* = 2.18; *X*^2^ = 4.47; *p* < 0.05) show a significant difference between the two talent groups. On the other hand, the ORs for the body weight points at a significant negative perspective (*OR* = 0.11; *X*^2^ = 6.69; *p* < 0.05) as the group of the heavier athletes have worse odds as the lighter counterparts.

The ORs for the soccer federation-, school-, and government-based talent promotion interventions were based on the binary code (Yes or No) for the participation in these campaigns. The OR for the diversity of competition participation in other sports before and besides soccer was based on the limit of at least three other sport activities. The chances for the youngsters to reach actually the maximum of soccer performance found in this study and thus to play particularly on the province level at the U17 age were substantially higher for those players who attended the local DFB soccer training center (*X*^2^ = 22.64; *p* < 0.001; η = 0.21). In contrast, the odds for the participants of the afternoon training course of the governmental talent promotion program (*X*^2^ = 2.15; *p* = 0.142) and for the members of the sport school classes (*X*^2^ = 2.52; *p* = 0.112) were not systematically better than those for the abstainers. In regard to the extent of diversity of the individual training activities in different competitive club sports, those children focusing solely on soccer or taking part in only one or two additional sport were assigned to group 0, whereas the children participating in three or even more competitive sport club activities before or besides their soccer engagement were assigned to group 2. The better odds for the multi-sport children (*X*^2^ = 3.96; *p* < 0.05; η = 0.09) are in line with the quota that three out of 13 children (23%) competing in at least three different club sports besides soccer reached the highest province level in soccer performance at the age of U17.

**Figure 7:**
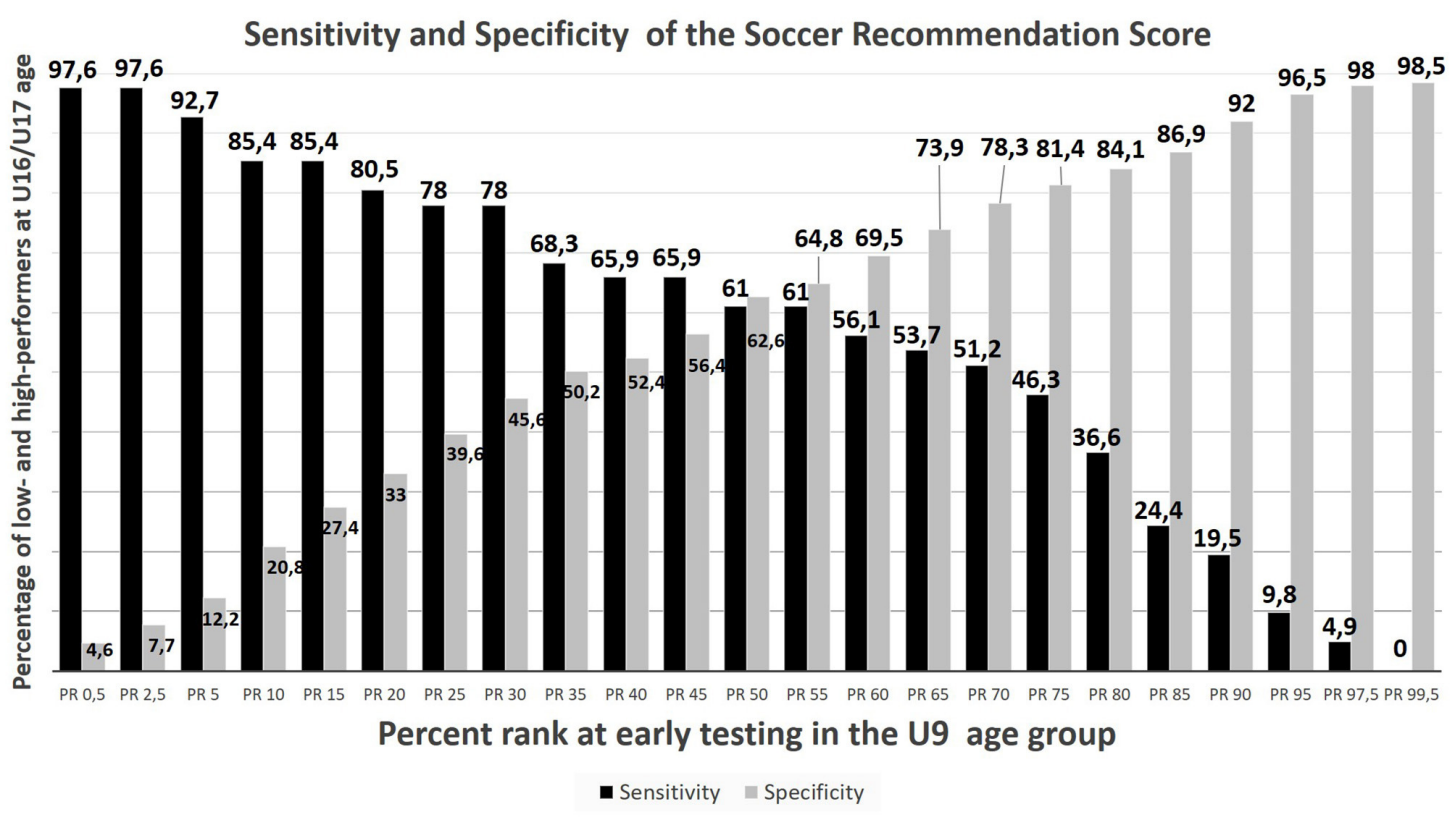
Specificity and sensitivity of the soccer recommendation score (SRS) of the U9 participants to predict a future soccer performance on the highest provincial level (level 5) at adolescence (U17).

In regard to the utility of the talent identification campaign, the questions of how many non-talented athletes will be disqualified (specificity of the testing) and how many talented youngsters will be successfully identified (sensitivity of the testing) by the test battery are of great interest (Hoener and Votteler, 2016; Bergkamp et al., 2019). Figure 7 documents that at a given cutoff limit of *z* = 1.0 (*PR* = 84) in the SRS, according to the sensitivity curve 24.4% of the participants, were correctly identified as future successful soccer players (true positives). The specificity of the testing amounts to 86.3%, which means that this fraction of low talented players (true negatives) would be excluded from talent promotion if the campaign aimed at securing at least a provincial soccer performance level. To optimize the balance between drafted talents and excluded non-talents, *Youden's J* of the SRS in Figure 7 documents that the best compromise is reached by a cutoff limit at PR 70 (*z* ≥ 0.5), where 51.2% of the future group of successful soccer players could be correctly identified, and 78.3% of the later-on less successful participants would be sorted out. In case of such a cutoff limit, the success rate in the group of the 120 drafted U9 talents would be 19.16%, as 23 of the draftees finally had reached the highest province level at age U17. Comparatively, the base rate in the tested 502 U9 soccer players was 7.97%, as a total of 41 talents reached the highest level in the future.

#### Regression Path Analysis

**Figure 8:**
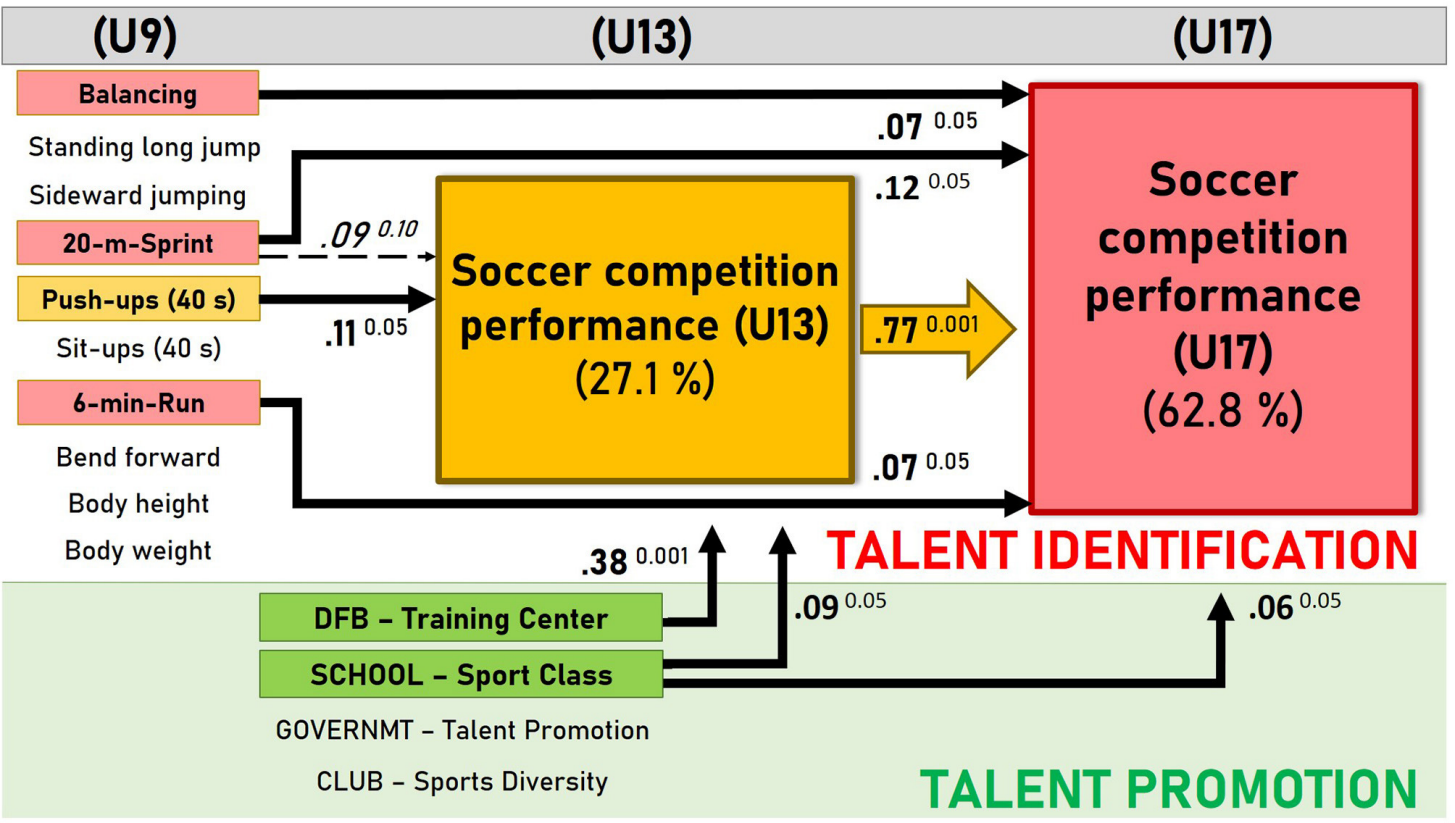
Regression path analysis of early performance characteristics at U9 age and their relevance for midterm performance development in youth and adolescent soccer players (*N* = 502).

Besides the prognostic validity of the early performance characteristics, the long-term influence of different talent promotion interventions is of major importance. In parallel to the testing campaign and besides the 3 weekly school sport lessons of 45-min duration in elementary and grammar schools, the youth athletes are subject to a variety of sport performance-enhancing measures. Thus, in a first step of the path analysis, we investigated not only the prognostic validity of the 10 physical, physiological, and coordinative performance characteristics but also the influence of the (i) diversity of the voluntary club sport participation and of the three official talent promotion interventions, (ii) governmental talent promotion program for the best movers, (iii) sport classes of the two local sport schools, and the (iv) training center of the German Soccer Federation. In the first step, also the U13 soccer performance was added to the list of regressors to predict the U17 soccer performance. In a second step, we left out the U17 soccer performance and substituted it by the U13 soccer performance. Now, the 10 performance characteristics and the four talent promotion measures served to predict the U13 soccer performance. Both regression analyses were tested for multicollinearity. Neither in the U17 regression model (0.492 < *TOL* < 0.976; 1.025 < *VIF* < 2.033) nor in the U13 model the tolerance (TOL) coefficients and variance inflation factors (VIFs), respectively (0.492 < *TOL* < 0.970, 1.024 < *VIF* < 2.033) confirmed such an intercorrelation between the independent variables that none of these predictors had to be excluded from one of the two combined models (Eid et al., 2017). Figure 8 shows the complete result of the regression-based path model documenting the statistical relevant pathways of influence of the predictors on the soccer performance development. The configuration of the model components in Figure 8 is based on the process structure of our abstract talent development model shown in the beginning (see Figure 1), thus adding the social talent promotion interventions from the sport-specific environment of the youth soccer players to the portfolio of the physical, physiological, and coordinative performance characteristics of the soccer athletes.

As can be seen in the path-analytical model in Figure 8, the adolescent soccer performance at age U17 [*F*_(15;463)_ = 54.83; *p* < 0.001] is predominantly determined by the intermediate soccer performance at age U13. Besides the intermediate soccer expertise at age U13, the adolescent soccer performance is also significantly influenced by early PF (20-m sprint and 6-min run; *p* < 0.05) as well as MC (dynamic balancing backward; *p* < 0.05). During the early stage of youth development, the intermediate U13 soccer performance [*F*_(14;464)_ = 13.71; *p* < 0.001] is primarily determined by the early MC at age U9, which is in particular represented by the difficult coordination of the upper limbs in the challenging push-up task (*p* < 0.05) and the sprint run performance (20-m sprint; *p* < 0.10), although the latter effect is smaller and only shows a tendency toward significance. The upper body coordination in the push-up test in the second graders shows a direct effect (beta = 0.11) on early soccer competition performance at age U13 and an indirect effect via this mediator on soccer competition performance at age U17. The indirect effect (beta = 0.08) reflects a full mediation because after controlling for the mediator, body coordination has no significant direct effect on soccer performance at age U17 (Preacher and Hayes, 2004). Finally, there was no systematic effect of the standing long jump, sideward jumping, abdominal strength endurance, torso flexibility, body height, or body weight on the soccer performance at early (U13) and late adolescent age (U17).

On the side of the talent promotion measures, a significant relation could be found between the membership in a sport class at school and the soccer competition performance of the children at age U13 (beta = 0.09; *p* < 0.05), and also at the later age U17 (beta = 0.06; *p* < 0.05). The highest effect is seen in the sport school support on the soccer success at age U13 (beta = 0.38; *p* < 0.001). On the other hand, no significant effect could be found between the engagement in the governmental afternoon sport course and the subsequent soccer performance at adolescent age. Also, in the total group, the number of additional club sport activities besides soccer was not related to the soccer competition performance at the ages U13 and U17. As only the players of the lowest and highest performance levels participated to a greater extent in more than one other sport besides soccer training, we obtained a significant curvilinear relation between the early diversity of club sport engagement and the future success in adolescent soccer competition [quadr *R*^2^ = 0.02; *F*_(2;497)_ = 5.90; *p* < 0.01].

## Discussion

It is imperative that early talent screening and sports orientation campaigns based on the assessment of the performance predictors and competition performance have to be related to a long-term talent development process and properly allocated to defined stages of a complex talent development model. Compared with recent studies (Huijgen et al., 2013; Hoener and Votteler, 2016; Hoener et al., 2017; Leyhr et al., 2018) investigating the prognostic relevance of mainly soccer-specific skills as talent predictors in male youth soccer, this study differs in that it assessed mainly the more general athleticism and MC of a very young male sample over a long-term period from the early talent screening in second grade (U9) until the gateway to expert performance at adolescent age (U17).

The present results are in line with Gil et al. (2007), who assumed only a short-term advantage of larger body dimensions but on the other hand contradict the later importance of the anthropometric variables body height and body weight found by Hoener et al. (2017) in early adolescence for more or less successful adult players. These findings could have resulted from two different factors. On the one hand, the elimination of the calendar age from our test data by partializing out the age (in months) from all test performance data could have led to a more homogeneous “age group.” On the other hand, there could exist a considerably lower relative age effect in the early talent development stage investigated in this study compared with middle or late adolescence (Musch and Grondin, 2001; Carling et al., 2009). Due to the early stage of the talent screening campaign, the U9 participants are still far from the pre-pubertal acceleration of the development of body dimensions, which might reduce the impact of the anthropometric predictors on the soccer performance of the young people. Nevertheless, to fully understand the relationship between body size and biological maturity (Meylan et al., 2010; Lago-Peñas et al., 2011; Lovell et al., 2015; Patel et al., 2019) and soccer performance development from childhood to adolescence, more differentiated analyses would be worthwhile.

According to the ANOVA results, and especially the ORs of the PF and MC tests, this longitudinal study verified the prognostic validity of all eight generic motor tests in the sport of soccer. In this context, the tendency of a significant long-term prognostic validity of the MC test balancing backward, as well as the explosive PF test standing long jump already at the U9 age, comes as no surprise (Reilly et al., 2000; Wong et al., 2009; Deprez et al., 2015; Sarmento et al., 2018) and might be an indicator of early athleticism that is relevant for soccer talents, be it innate or a specific outcome of early soccer training experience (Franks et al., 1999). All in all, at least the design of our study is comparable with the longitudinal approach of Hoener and Votteler (2016) and Leyhr et al. (2018), although their studies analyzed the prognostic relevance of soccer-specific motor diagnostics on the talent development stage from early adolescence (U12–U15) until junior age (U16–U19). In contrast, their study focused on elite youth athletes, having already excelled during early, mid, and late adolescence. Although our study was based on more general soccer performance prerequisites, it might help to complete the picture of the soccer performance development reaching from the search for an early soccer-related profile of general athleticism in a very heterogeneous sample at elementary-school age until the soccer-specific talent selection measures at junior age, which are normally based on very homogeneous soccer populations and include high-fidelity diagnostics (Bergkamp et al., 2019). In line with our results, Deprez et al. (2015), Figueiredo et al. (2009), Gonaus and Mueller (2012), Le Gall et al. (2010), and Zuber et al. (2015, 2016) underlined the midterm (≥2 years) relevance of running endurance and sprint speed in youth soccer players. Interestingly, in our study, the 20-m sprint and the 6-min run performances were systematically correlated (*r*_tt_ = 0.39; *n* = 484; *p* < 0.001), which might refer to an age-specific, general running ability. In regard to the direct pathway between balancing and later soccer performance, Mirkov et al. (2010) likewise stressed the high relevance of coordination for future success in soccer. Another reason could be that children who already played soccer before the testing have improved their motor coordination and, thus, achieved better test results. Last but not least, also the participation of 70 soccer athletes in the different talent promotion programs, which stress especially general coordination ability and fundamental movement skills, might have contributed to the long-term impact of dynamic balance on the soccer performance. As a consequence of the particular results in the OR and the path-analytical model, the composition of the SRS should be revisited. It seems that a supplement of the highly valid dynamical balance test could contribute to a better soccer talent forecast at least at the elementary-school age, which would be in line not only with the findings of Mirkov et al. (2010) but also with the test ranking of the coaches obtained before.

The structure of the path-analytical model outlined in Figure 8 was based on our process-oriented talent development model described in the beginning (see Figure 1) and is consistent with the general model of talent development outlined by Abbot and Collins (2002), Fisher and Bailey (2008), and Heller and Hany (1986), where the performance-enhancing effect of the talent promotion environment is taken for granted. It is well-known and in line with our findings that elite sport schools (Granacher and Borde, 2017), as well as the talent promotion training centers of the German Soccer Federation (Hoener et al., 2015), have contributed largely to performance development and sporting success. On the other hand, we could not confirm a positive effect of the province-wide talent promotion campaign “Talent Search and Talent Promotion” of the regional government on the performance of youth soccer players. The lack of evidence might be due to the early-onset, low 90-min per week volume and short duration of the campaign between ages 6 and 10, as well as the generic training program focusing predominantly on fundamental movement skills. So our result is not surprising, as the objectives of this program are not to foster soccer-specific skills but more general PF and MC independent from the children's decision on the talent development pathway. As coach-led, soccer-specific practice necessary for later success (Haugaasen et al., 2014; Hornig et al., 2014) does not happen in this course, it might exert only little effect on future soccer expertise at adolescent age.

Our findings were not fully consistent in regard to the controversial debate of whether a high diversity of early club sport activities enhanced talent development by a greater portfolio of protective coordination patterns and transferable technical skills (Cote and Hay, 2002; Baker, 2003; Baker et al., 2003; Soberlak and Cote, 2003; Cote et al., 2009; Myer et al., 2015) contributes to better learning pre-requisites to optimize soccer-specific technical skills (Huijgen et al., 2010, 2013) or has more negative than positive consequences for long-term athletic development by impairing sport-specific technical and tactical education due to reduced time, energy, and logistic resources (Helsen et al., 1998; Ward et al., 2007; Forsman et al., 2016a; Zuber et al., 2016). In our path-analytical model, the contribution of the diversity of club sport participation did not play a significant role, as in the total group of all soccer players there was no significant linear correlation with the performance levels reached at adolescence. In general, our path-analytical model confirmed the findings of Ford et al. (2009), Ford and Williams (2012), and Ward et al. (2007), as it did not underline a positive effect of an early diversification of club sport participation. On the other hand, at least the ORs of the diversified club sport participation were in line with Guellich et al. (2017) that such a multi-sport engagement could serve as a promising strategy to reach the highest province soccer performance level later on. The explanation for the missing relation might lie in the fact that only in the lowest and highest performance groups of the soccer players a major fraction of children engaged in three or even more competitive sports. In the medium soccer performance groups of the district or county performance levels (2–3), most of the youth athletes concentrated solely on soccer or participated in one or two additional sports only. Our interpretation for this situation is that at the low soccer performance level, a substantial number of athletes with good general performance characteristics preferred other sports as main disciplines and thus participated in soccer training and competition games just as side events to their main sport. This interpretation is in line with the significant quadratic bivariate regression function found between the training diversity that is the number of sports performed during youth development and the adolescent soccer performance level reached at adolescence. Nevertheless, we assume that the relation between the diversity of early club sport engagement and future soccer success again disappears when the best youth soccer athletes performing on national or international youth level were included, as the findings of Ford et al. (2012) and Hornig et al. (2014) suggest. In our study, all of the province level soccer athletes practiced in the three sports—track and field, tennis, and table tennis only—which is in line with the specialized sampling approach of Sieghartsleitner et al. (2018) postulating that additional side-sport activities should show at least complementary relations and thus provide positive contributions to the main sport soccer to enhance the specific PF or MC profile needed there.

There are some limitations in this study. The operationalization of the individual soccer performance by means of the highest soccer performance level reached by the club team is still debatable (Baker et al., 2015; Bergkamp et al., 2019; Leyhr et al., 2020). To compensate for the lack of individual in-match player performance assessment, we introduced an additional control step to have the individual soccer performance criterion finally checked fine-grained player-per-player by the regional talent coordinator of the German Soccer Federation (DFB). Besides that, we can also assume that due to natural selection and club policies on the higher U17 county and province levels, the individual performances correspond pretty close to the teams' overall soccer performance. Nevertheless, there might be some internal validity problems at least on the lower levels 1–2, which are on a local level. Here, the player participation in a youth club team might also depend to a certain degree on the preference of early peer relations or shorter travel distances to the training and competition sites and thus underestimate the individual soccer performance level. Thus, on the two lowest levels, team success might not fully represent the individual athletes' performance capacity. Furthermore, due to less frequent reporting on individual player participation in the media and internet match reports on the lowest levels 1–2, the range in the criterion soccer performance could be somewhat restricted, so that the pathways between the predictors and the later-on soccer performances found in our study might be underestimated (Bergkamp et al., 2019). Especially, in our path-analytical model, more precisely classified low-level participants could improve the significance and prognostic validity of the different performance characteristics of the U9 players. Besides the low fidelity of the criterion variable team performance level, a limitation of our study is also that the investigated performance levels end up at the sub-elite Hessen province level. From a nationwide perspective on talent identification, only the highest youth soccer performance levels of the U17 Regionalliga (level 6) and Bundesliga (level 7) truly represent youth elite soccer performance. So it has to be stressed that there is still a gap between the soccer performance investigated in this study and the nation's best soccer performances within the U17 age group. In the U9 age group, the generic test battery used here generally allows for a first comprehensive performance diagnosis to identify good movers with a suitable profile of anthropometry, PF, and MC for a deliberate soccer training. Besides the initial quality of the performance characteristics other factors like sport-specific training initiation and soccer experience, learning conditions and facilities, quality of training and training volume, and field position might influence the performance development at youth age. So the lack of information about these potentially confounding variables is a limitation of the present study.

Also, there is still room for improvement in the game sports where test items focusing on technical skills and agility without or in combination with decision-making could be added to improve the prognostics (O'Connor et al., 2016; Ulbricht et al., 2016; Schorer et al., 2017). On the one hand, such predictors of higher fidelity may lead to a better explanation of the variance between the soccer performance groups (Bergkamp et al., 2019). On the other hand, in soccer, Hohmann et al. (2018) observed at elementary-school age a lower prognostic validity of sport-specific skill tests compared with generic physical or physiological tests, which the authors attributed to the higher difficulty of technical tests and the short time span of soccer-specific education. In order to do justice to the multidimensional character of football performance (Huijgen et al., 2010), in future studies at primary school age, the general tests should be supplemented, if not by football-specific tests, then at least by semi-specific tests to test fundamental ball-handling skills (Rylander et al., 2019). Despite such concerns, it is obvious that research has to be extended to the early age group of elementary-school children, as Ford et al. (2020) have reported recently that a substantial fraction of worldwide youth soccer academies have installed talent identification systems at the age group level of 8 to 11 year-old players already. In addition to sport-specific test extensions, psychological test items might also provide further indications of potential future top performers (Morris, 2000; Epuran et al., 2008; Forsman et al., 2016a; Johnston et al., 2018; Murr et al., 2018a,b), although it is still unclear whether such attempts could be a fruitful endeavor at the early stage of talent development during elementary-school age already. At least in the U12 age group, Zuber et al. (2015), as well as Hoener et al. (2015), have made promising attempts to integrate psychological testing in a talent identification campaign of the Swiss Soccer Federation (SFV) and the German Soccer Federation (DFB), respectively. All in all, further research is needed not only to contribute to the extension of the current knowledge on the prognostic validity of motor assessment on this early talent development stage but also to clarify whether such general talent diagnostic campaigns are suitable to support youth sport practitioners in the task of recommending and assigning the right sport to each of the promising individuals (Pion, 2015).

Nevertheless, despite multifaceted and optimized test tasks as well as sophisticated multivariate statistical tools, not every talented athlete can always be surely identified, be it at elementary-school age or during puberty and adolescence. In contrast to linear talent prediction methods, some unexpected players develop into professional athletes, as even during late adolescence very few career paths are straightforward (Elferink-Gemser et al., 2011; Gulbin et al., 2013; Li et al., 2018). Thus, very often unsteady development of performance characteristics (Vaeyens et al., 2008; Fransen et al., 2017) and non-linear pathways to the top of the scene are determined by many interacting dynamic parameters to which each athlete reacts in a unique way (Phillips et al., 2010), and which should be reflected in the research methodology as well (Den Hartigh et al., 2016; Fransen et al., 2017; Leyhr et al., 2020).

## Conclusion

Our study shows that early talent screening and sports orientation can be used to make valid statements in regard to future success of young soccer players. It also provides reliable empirical knowledge on the prognostic relevance of eight generic PF and MC tests in a regional talent screening and sports orientation campaign. The results show motor predictors' prognostic validity over a long-term period (on average about 8 years) after controlling all test data for calendar age. The specificity of the generic testing in the second grade is very high, and the majority of non-talents is advised to pursue sports other than soccer for which they were better suited. However, due to the medium sensitivity of the testing, recommending elementary-school children to focus solemnly on the sport soccer still remains a very complex practical and theoretical problem. The practical application of the test battery assessing speed, endurance, and coordinative abilities turned out to be a useful tool for talent orientation as a combination of multifaceted testing and a subsequent sports recommendation on the basis of the best match of the individual profile with the concrete demands of specific sports like soccer.

Further studies with alternate methodological approaches, like person-oriented pattern analyses (Zuber et al., 2015, 2016; Zibung et al., 2016), and a promising combination of linear and non-linear prognostic tools (Pion et al., 2016) should be further examined and compared with each other to identify the corresponding strengths and weaknesses. In doing so, the research on talent orientation may provide coaches with more scientifically valid tools for supporting their talent development strategies as well as offering a deeper understanding of the effectiveness of talent promotion interventions in the process of the long-term development of talented youth soccer players.

## Data Availability Statement

The data associated with the paper are not publicly available but are available from the corresponding author on reasonable request.

## Ethics Statement

The studies involving human participants were reviewed and approved by Municipality of Fulda, University of Bayreuth. Written informed consent to participate in this study was provided by the participants' legal guardian/next of kin. The Declaration of Helsinki was checked and adhered to in all parts of this study.

## Author Contributions

AH contributed on to the design and implementation of the research. MS performed the test data collection. All authors contributed to the final version of the manuscript.

## Conflict of Interest

The authors declare that the research was conducted in the absence of any commercial or financial relationships that could be construed as a potential conflict of interest.

## Footnotes

^1^The predictive validity of the age-specific soccer performance variables even increased with age, as was shown in the oldest fraction of the investigated sample: U13 ^*^ U19: *r*_tt_ = 0.87, *n* = 140, *p* < 0.001; U15 ^*^ U19: *r*_tt_ = 0.70, *n* = 140, *p* < 0.001; U17 ^*^ U19: *r*_tt_ = 0.97, *n* = 140, *p* < 0.001.
